# Analysis and insights into recombination signals in lumpy skin disease virus recovered in the field

**DOI:** 10.1371/journal.pone.0207480

**Published:** 2018-12-12

**Authors:** Alexander Sprygin, Yurii Babin, Yana Pestova, Svetlana Kononova, David B. Wallace, Antoinette Van Schalkwyk, Olga Byadovskaya, Vyacheslav Diev, Dmitry Lozovoy, Alexander Kononov

**Affiliations:** 1 Federal Center for Animal Health, Vladimir, Russia; 2 Federal Budget Institution of Science "Central Research Institute of Epidemiology”, Moscow, Russia; 3 ARC-Onderstepoort Veterinary Research institute, Onderstepoort, South Africa; 4 Department of Veterinary Tropical Diseases, Faculty of Veterinary Science, University of Pretoria, Onderstepoort, South Africa; Oklahoma State University, UNITED STATES

## Abstract

Wide spread incidences of vaccine-like strains of lumpy skin disease virus (LSDV) have recently been reported in a Russian region with a neighboring country that actively vaccinate with a live attenuated LSD vaccine. The use of live-attenuated viruses (LAVs) as vaccines during an active outbreak, creates potential ground for coinfection of hosts and emergence of a strain combining genetic fragments of both parental vaccine and field strains. In this study, we analyse the vaccine-like strain LSDV RUSSIA/Saratov/2017 detected in Saratovskaya oblast, a region sharing border with Kazakhstan. To gain insight into possible recombination signals, a full-genome next-generation sequencing of the viral genome was performed using the Illumina platform. The genome contains the backbone of a live-attenuated vaccine with a patchwork of wild-type field virus DNA fragments located throughout. A total of 27 recombination events were identified. The average distance between the recombination sites was 3400 base pairs (bp). The impact of the recombination events on the virulence and transmission capacity of the identified virus remains to be clarified. These findings provide evidence for the first time of genetic exchanges between closely related strains of capripoxviruses in the field and a vaccine strain, and prompt a revisiting of the vaccination issue for a safe and efficacious prevention and control strategy of LSD.

## Introduction

Lumpy skin disease (LSD) was first described as “pseudo-urticaria” in Zambia in 1929 [1]. It was identified in the 1940s as an infectious disease caused by lumpy skin disease virus (LSDV), rather than hypersensitivity to insect bites or plant poisoning [2]. The onset of skin lesions following summer rainfalls, when insect abundance is at its greatest, resulted in the rapid spread of the disease throughout southern Africa, establishing it as an endemic disease in this sub-region [3]. The disease was thought to be confined to southern Africa, until its rapid spread into Central and Eastern Africa by 1956 and subsequently in 1989 it expanded beyond the African continent and spilled over into the Middle East [4]. From 2015, LSD outbreaks were documented in Turkey, the EU and Russia [5].

LSDV appears to be mechanically vectored by blood-sucking arthropods such as stable flies, mosquitoes and hard ticks. The genome has also been identified in *Musca domestica* flies, but the clinical relevance of this finding remains obscure [6]. The fact that LSD tends to occur at periods of greatest arthropod activity (warm, wet months) suggests that different vector species may play a role in virus transmission under these climatic conditions.

A single immunological type of LSDV with the “Neethling” strain as prototype has been described [7]. The virus belongs to the genus *Capripoxvirus*, in the family *Poxviridae* and its genome is represented by double-stranded DNA enclosed in a lipid envelope. The genome is approximately 151 thousand base pairs (bps) in size, encoding 156 putative genes [8]. Evidence based on restriction enzyme fragment length polymorphisms (RFLPs) indicated that a subset of capripoxviruses were derived from an ancestral strain through genetic recombination with other naturally occurring capripoxviruses in the field [9]. Despite the numerous reports of genetic recombination between the genomes of orthopoxviruses, detected by selection or screening viruses in laboratory animals or cell culture, published examples of poxvirus recombinants clearly arising in the field are still lacking [10]. In this regard, Gershon (1989) raised concerns on the likelihood of recombination between a vaccine and a naturally occurring poxvirus. Moreover, the possibility of recombination between co-infecting poxviruses has already been established under laboratory conditions [11].

Live attenuated LSD vaccines have been in use in Africa for over 50 years and are now widely used in most affected northern hemisphere countries (e.g. Serbia, Croatia, Kazakhstan and Armenia). This creates a potential hazard arising from the emergence of a novel LSDV strain combining both properties of parental attenuated and field strains should co-infection occur; however the risk of such recombination events occurring are currently unknown and unpredictable with the probability of occurrence deemed very low mainly due to a lack of full genome sequences of LSDV available. The emergence of mosaic viruses deriving from the use of live vaccine in the face of field strain circulation has already been shown [12, 13].

Due to the intrinsic features of poxvirus “factory” formation during replication in the cytoplasm [11], hybrid progeny are not generated in high abundance. As for capripoxviruses, no conclusive evidence of recombination within the genus has yet been obtained in the field.

The territory of the Russian Federation first experienced an incursion of LSD in 2015 in Northern Caucasus, which then swept through a vast geographic area in the European part of Russia in 2016 [14]. Despite successful efforts to contain the disease, in 2017 its spread pattern changed in terms of geography to regions which opted out of using vaccination [15]. However, amongst the LSDVs obtained during the documented outbreaks in 2017 we discovered the circulation of a vaccine-like strain linked to LSD outbreaks [6]. This is of major concern since the use of vaccines based on live-attenuated LSDVs is prohibited across the territory of the Russian Federation, where only heterologous preparations based on sheeppox virus are allowed for safety reasons.

This study is the first report of a complete genome sequence of a recombinant vaccine-like LSDV obtained from the field.

## Materials and methods

### Viral strain

The vaccine-like LSDV analysed in the study (designated LSDV_RUSSIA/Saratov/2017) was recovered in Saratovskaya oblast (Altata settlement, latitude 51.10, longitude 48.72) in 2017 from a cow exhibiting severe clinical signs of LSD which included fever, skin lesions (coalescing together) and a decrease in milk production.

### PCR analysis

The presence of LSDV was confirmed following an initial PCR assay analysis targeting the conserved P32 gene fragment of capripoxviruses [16]. Subsequently, the sample was subjected to classical PCRs differentiating between vaccine-like viruses and wild-type field isolates based on GPCR and RPO30 genes [17, 18] and real-time PCR directed against LSDV005 of the vaccine strains [6] and LSDV127 of field isolates (the EEV gene with a 27 bp insertion unique to field strains only) [19].

### Virus propagation and purification

The virus was cultivated in primary lamb testis cells and harvested when 85% of the cells exhibited cytopathic effect. To release virus into the media, infected monolayered cells were frozen and thawed three times, followed by centrifugation at +4 °C.

### DNA extraction

Total DNA was extracted using the DNeasy blood and tissue kit (Qiagen, Germany), according to the manufacturer’s instructions. The final elution was conducted with nuclease-free water. A sequencing library was constructed using the Nextera XT DNA library preparation kit and sequencing was performed using a MiSeq reagent kit version 2 with 2 × 250-bp paired-end sequencing on a MiSeq benchtop sequencer (Illumina, USA).

### Sequence assembly and alignment

In order to assemble the genome, reads were mapped to the reference genome (Neethling 1959, AF409138). Total reads were further mapped to the newly assembled virus genome, designated “LSDV RUSSIA/Saratov/2017”, with an average coverage depth of 45× and an average map length of 250 nucleotides (nt). The genome consists of 153844 nt, with 156 predicted protein-coding genes. Open reading frames were predicted using GATU software [20]. The full genome sequence of LSDV Saratov/2017 was deposited in GenBank database under MH646674.

### Genome and phylogenetic analysis

**Fig 1:**
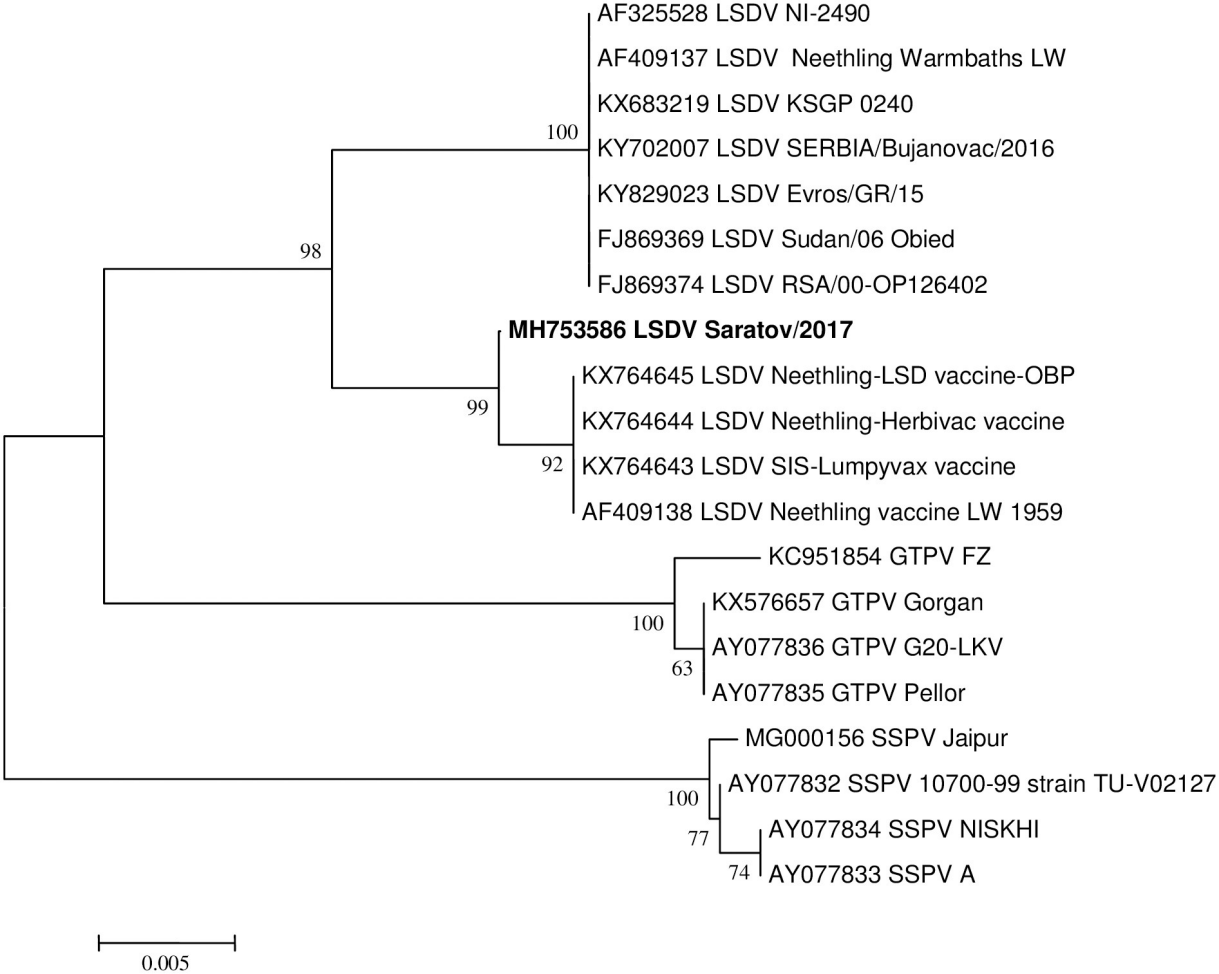
Neighbor joining tree showing phylogenetic relationship based on the GPCR region for LSDV RUSSIA/Saratov/2017.

Complete genome sequences of field and vaccine lumpy skin disease viruses (LSDV), sheep pox viruses (SPPV) and goat pox viruses (GTPV) were obtained from GenBank (www.ncbi.nlm.nih.gov). An alignment of the selected sequences with the consensus sequence of LSDV RUSSIA/Saratov/2017 was performed using the ClustalW algorithm in BioEdit Sequence Alignment Editor with default parameters. The alignment was used to infer the evolutionary history of the pox viruses by using the Maximum Likelihood method based on the General Time Reversible model [21]. The tree with the highest log likelihood (-242517, 1430) is shown (Fig 1). The percentage of trees in which the associated taxa clustered together is shown next to the branches. Initial tree(s) for the heuristic search were obtained by applying the Neighbor-Joining method to a matrix of pairwise distances estimated using the Maximum Composite Likelihood (MCL) approach. A discrete Gamma distribution was used to model evolutionary rate differences among sites (4 categories [+*G*, parameter = 0,1000]). The tree is drawn to scale, with branch lengths measured in the number of substitutions per site. The analysis involved 19 nucleotide sequences. There were a total of 153844 positions in the final dataset. Evolutionary analyses were conducted in MEGA6 [22].

### Recombination analysis using RDP4

An alignment consisting of ten LSDV complete genomes was constructed as previously described and subjected to analysis using a bootscan/rescan recombination test [23], MAXCHI [24], GENECONV [25], CHIMAERA [26] and the SISCAN method [27] within the RDP software package (v4.39) with default settings, to identify potential intra-segment recombination events [28]. Potential recombinants were further characterized using SimPlot [29].

### Ethics statement

All experimental procedures were reviewed and approved by FGBI ARRIAH Animal Ethics Committee.

## Results

### Virus recovery and culturing

LSDV RUSSIA/Saratov/2017 was obtained from a backyard cow exhibiting clinical signs consistent with LSDV infection. Skin lesions, fever and a decrease in milk production were observed. Samples were collected and the animal was culled, the carcass immediately destroyed and the remains were safely disposed of. Skin lesions were aseptically transferred to the FGBI ARRIAH for virus isolation.

### Phylogenetic analysis

**Fig 2:**
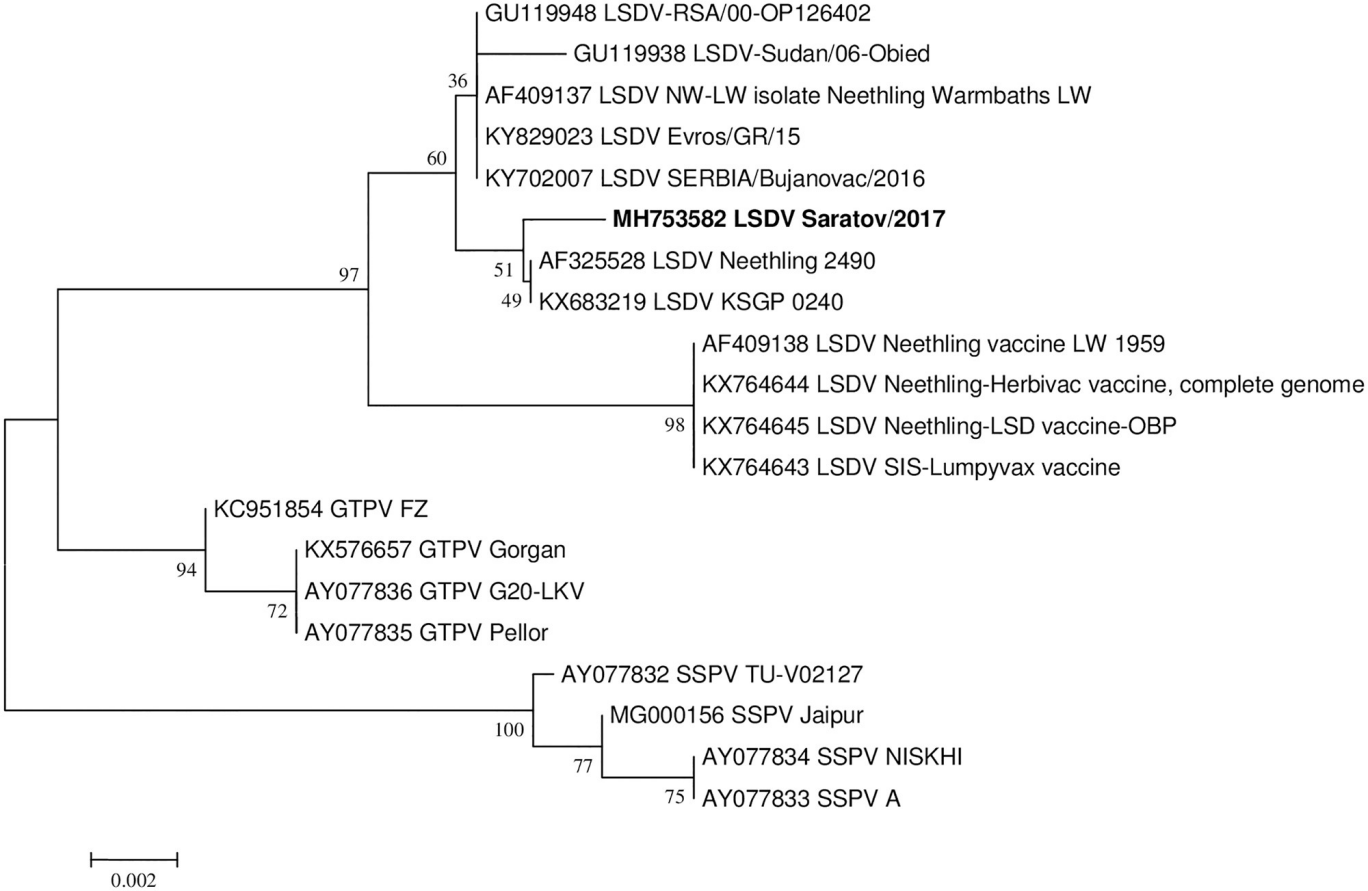
Neighbor joining tree showing phylogenetic relationship based on the RPO30 region for LSDV RUSSIA/Saratov/2017.

The sample tested positive for capripox DNA based on the P32 gene [15]. The Sprygin et al vaccine real-time PCR assay [6] and field assay based on LSD126 [15] identified neither vaccine nor field virus. Whereas the sequencing based on GCPR and RPO30 targets provided incongruent phylogenies for the strain (Figs 1 and 2). Sequence analysis of the GPCR region cluster LSDV RUSSIA/Saratov/2017 with the live attenuated vaccine or vaccine-derived viruses (Fig 1), while analysis of the sequence data obtained from the RPO30 region cluster the same LSDV RUSSIA/Saratov/2017 with the wild type field viruses (Fig 2). In order to exclude a contamination as a possible reason for the inconsistency in the phylogenetic clustering, at least 22 clones of both targets were sequenced. Each of these clones were identical and the representative sequences of RPO30 and GPCR of LSDV RUSSIA/Saratov/2017 were deposited in GenBank under MH753582 and MH753586, respectively. In order to resolve incongruence between the phylogenies, the complete genome sequence of the virus was determined and the possible evolutionary history of the virus inferred.

**Fig 3:**
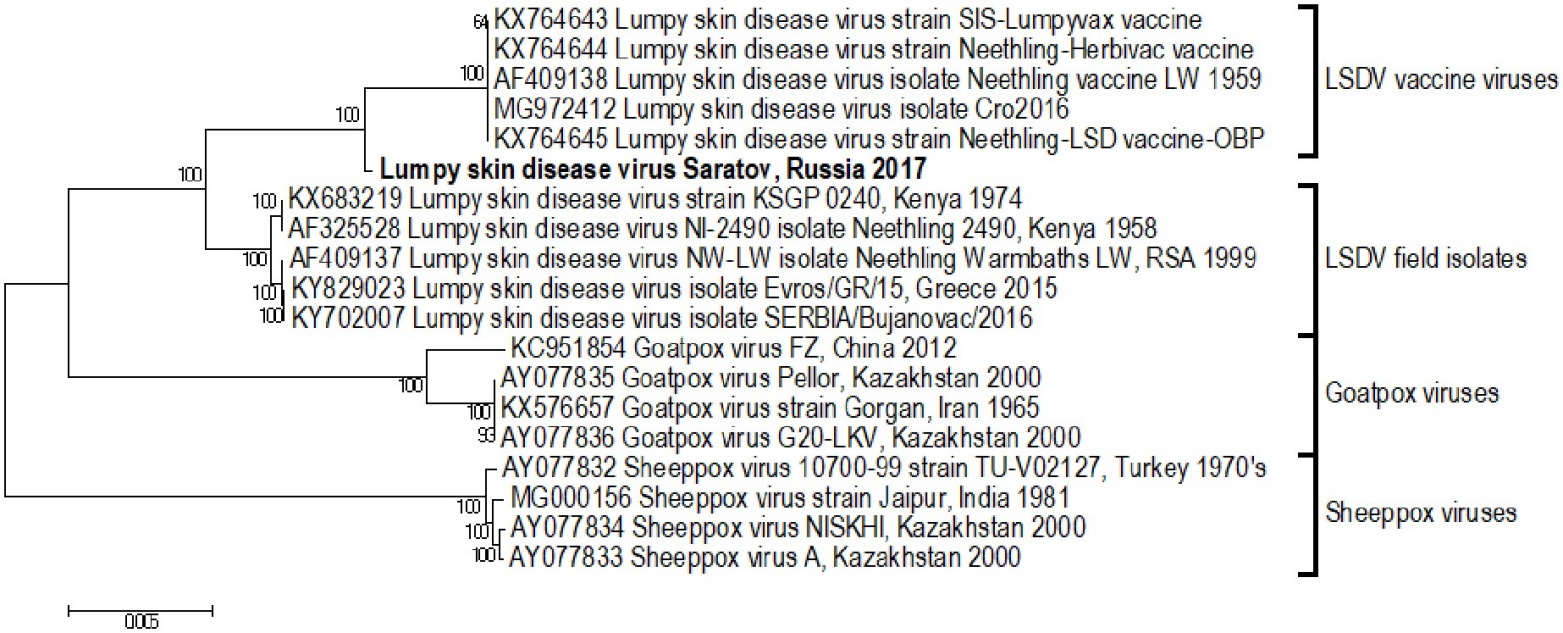
Phylogenetic comparison of 19 capripoxvirus full genomes. Distinct clusters separating LSD, SPP and GTP viruses are clearly evident (isolate LSDV RUSSIA/Saratov/2017, in bold).

The evolutionary relationship of LSDV, SPPV and GTPV was evaluated using a maximum-likelihood phylogenetic tree (Fig 3). It is evident that the genomes of the three members of the genus are highly conserved, with more than 97% sequence identity. The LSD viruses cluster within two distinct groups, the first composed of vaccine viruses and the second of field isolates (Fig 3). Isolate LSDV RUSSIA/Saratov/2017 clusters between the vaccine and field virus groups, prompting an investigation into the possible effect of intragenic recombination on the origin of the genome of this isolate.

### Recombination analysis

**Fig 4:**
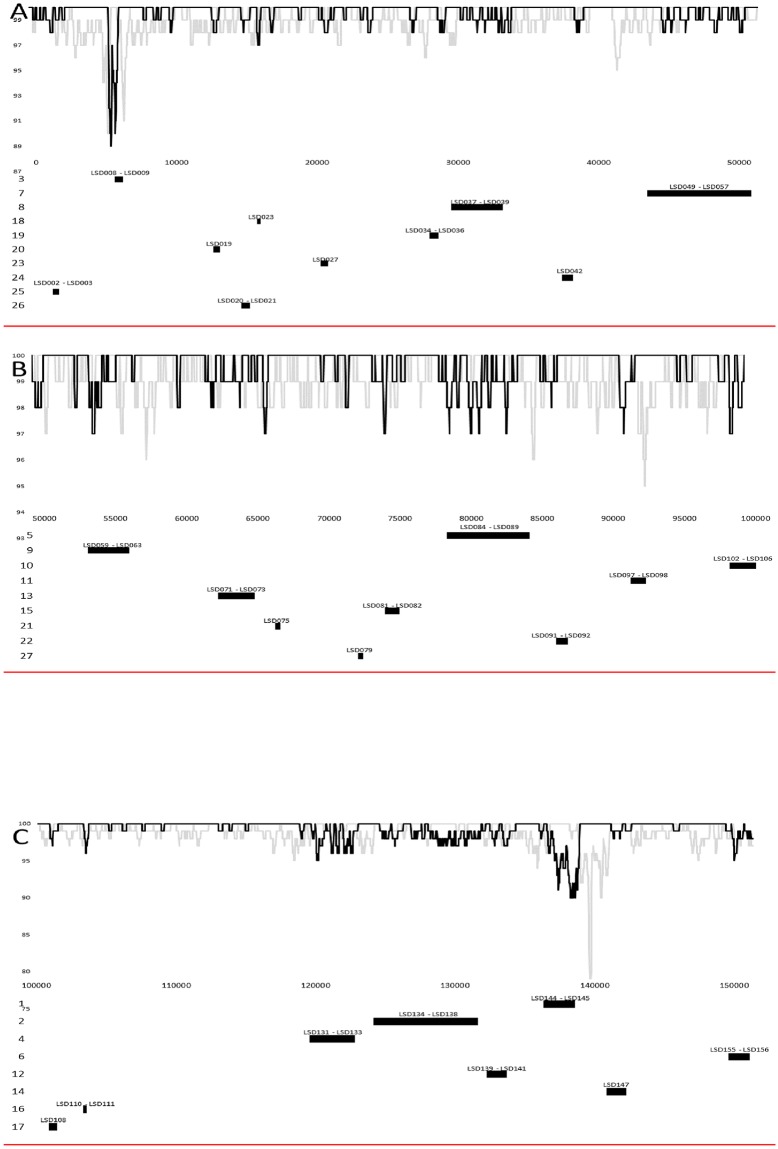
Percentage sequence similarity between SIS-Lumpyvax_vaccine (KX764643) in black and Bujanovac/Serbia/2016 (KY702007) compared to the genome of virus RUSSIA/Saratov/2017, using SimPlot. Graphical representation of the size and position of the predicted recombination events on the LSDV genome at the bottom of the similarity plot. The proteins affected by the recombination events are indicated on the top and the size of the recombined region at the bottom. Graphical representation of (A) basepair 1 to 50,000 (B) base pair 50,001 to 100,000 and (C) base pair 100,001 to 151,000 of the LSDV virus RUSSIA/Saratov/2017 genome.

The LSDVs clustering within the vaccine or vaccine-derived group (SIS-Lumpyvax, Herbivac, OBP vaccine and isolate Cro2016) share more than 99.9% sequence identity, while the viruses within the field isolate group cluster (Kenya KSGP0240, Kenya NI-2490, RSA Warmbaths, Greece Evros/GR15 and Serbia/Bujanovac/2016) share more than 99.8% sequence identity. All nine previously published virus genomes have between 98.2 and 98.4% sequence identity between each other over the 151kb genome. In contrast, isolate LSDV RUSSIA/Saratov/2017 is more closely related to the vaccine viruses (>99.3%) than the field isolates (>98.64%). This indicates that LSDV RUSSIA/Saratov/2017 has a genome backbone similar to a vaccine or vaccine-derived virus. In this case we have no evidence to suggest a specific vaccine strain or particular manufacturer to assign the genetic backbone of LSDV RUSSIA/Saratov/2017 to, due to the overall high percentage sequence identity of the vaccine group. Evidence for intragenic recombination was determined through use of the different methods described in the RDP4 package. A total of 27 recombination events were predicted and are listed in Table 1 or are graphically presented in Fig 4.

**Table 1:** Predicted recombination events using different detection methods within the RDP4 program. The 27 predicted recombination events and their breakpoint positions within the alignment are indicated. The size of the recombination region is determined based on the size of the recombinant sequence. RUSSIA /Saratov /2017 was either predicted as the recombinant sequence and the vaccine group as the major parent sequence or reversed for one event, while in all the events the field group of viruses were the minor parent. The number of base pair differences between the recombinant sequence and the minor parent sequence is calculated for the region mentioned and similarly the number of nucleotide differences (bps) between the recombinant sequence and the major parent. The differences in base pairs between the major and minor parents are indicated. Statistical support generated through the different detection methods are provided for each of the recombination events.

RDP Event Number	Breakpoint Positions in Alignment		Size of predicted recombination region (bp)	Predicted recombination region				Detection Methods					
	Begin	End		Nucleotide differences (recombinant to minor parent(s))	Nucleotide differences (recombinant to major parent(s))	Nucleotide differences (minor parent(s) to major parent(s))	Protein(s)	RDP	GENECONV	Bootscan	Maxchi	Chimaera	SiSscan
**1**	136364	138545	2176	3–7 bp	124 bp	124–126 bp	LSD144—LSD145	1,52E-48	8,81E-45	3,76E-47	3,52E-26	4,88E-26	2,88E-28
**2**	124188	131635	7442	11–16 bp	105–106 bp	111–118 bp	LSD134—LSD138	3,28E-32	1,37E-33	6,38E-31	8,75E-05	8,58E-05	2,15E-18
**3**	5614	6169	555	3 bp	43 bp	46 bp	LSD008—LSD009	9,96E-23	5,14E-20	9,51E-23	4,94E-13	4,00E-13	NS
**4**	119575	122787	3204	17–19 bp	71 bp	83–86 bp	LSD131—LSD134	1,65E-22	2,86E-16	1,22E-09	4,79E-14	9,83E-14	NS
**5**	78289	84100	5809	4–8 bp	52 bp	56–57 bp	LSD084—LSD089	9,32E-20	6,06E-24	1,71E-19	8,27E-11	5,56E-11	2,06E-11
**6**	149628	151098*	1230	7–8 bp	21–22 bp	22 bp	LSD154—LSD156	1,14E-19	3,52E-16	1,66E-18	8,80E-06	NS	NS
**7**	43456	50790	7331	12–15 bp	38 bp	50–51 bp	LSD049—LSD057	1,88E-16	3,97E-11	3,00E-13	2,15E-11	4,64E-12	2,01E-07
**8**	29524*	33134	3609	0–2 bp	21–22 bp	20 -22bp	LSD035—LSD039	1,64E-13	7,65E-13	1,63E-13	2,11E-07	2,08E-07	NS
**9**	53041*	55902	3041	2–6 bp	22 bp	24–26 bp	LSD059—LSD063	1,03E-12	2,96E-11	1,42E-12	4,74E-07	4,69E-07	1,80E-05
**10**	98171*	100057	1885	6–10 bp	19 bp	23–25 bp	LSD102—LSD106	3,56E-08	1,07E-04	3,16E-02	9,30E-03	9,19E-03	NS
**11**	91227	92254	1027	No difference	12 bp	12 bp	LSD097—LSD098	NS	5,96E-07	5,75E-08	3,26E-03	7,22E-03	NS
**12**	132260*	133690	1430	7–10 bp	19 bp	26–28 bp	LSD139—LSD141	2,84E-08	1,59E-03	1,73E-03	2,38E-04	2,35E-04	0,014247
**13**	62209*	64726*	2517	6 bp	17 bp	21 bp	LSD071—LSD073	4,40E-08	7,46E-06	8,66E-07	3,27E-05	2,82E-05	NS
**14**	140868	142259*	1391	No difference	10 bp	10 bp	LSD147	NS	1,04E-06	3,49E-07	NS	NS	NS
**15**	73969	74922*	953	No difference	9 bp	9 bp	LSD081—LSD082	NS	1,61E-05	1,95E-06	NS	NS	NS
**16**	103339*	103572	233	0–1 bp	7 bp	7–8 bp	LSD110—LSD111	NS	1,54E-03	2,15E-04	NS	NS	NS
**17**	100891	101452*	561	0–1 bp	7 bp	6–7 bp	LSD108	NS	1,10E-03	1,51E-04	NS	NS	NS
**18**	15713*	15921	208	No difference	6–7 bp	6–7 bp	LSD023	NS	5,08E-04	1,33E-02	NS	NS	NS
**19**	27981	28590	608	0–2 bp	7 bp	6–7 bp	LSD034—LSD036 (LW035a)	NS	5,32E-04	1,02E-03	NS	NS	NS
**20**	12602	13041	439	No difference	6 bp	6 bp	LSD019	NS	3,03E-03	5,80E-04	NS	NS	NS
**21**	66209*	66566*	357	No difference	6 bp	6 bp	LSD075	NS	2,18E-03	4,11E-04	NS	NS	NS
**22**	85973	86804	831	0–1 bp	6 bp	6–7 bp	LSD091—LSD092	NS	1,75E-03	3,24E-04	NS	NS	NS
**23**	20245*	20701	456	3–4 bp	6 bp	9–10 bp	LSD027	NS	2,20E-02	2,61E-04	NS	NS	NS
**24**	37425*	38121	696	0–2 bp	6 bp	4–6 bp	LSD042	NS	1,08E-03	1,74E-04	NS	NS	NS
**25**	1223*	1611	387	0–1 bp	3 bp	3–4 bp	LSD002—LSD003	NS	NS	3,95E-03	NS	NS	NS
**26**	14618*	15180	562	1–2 bp	6 bp	7–8 bp	LSD020—LSD021	NS	NS	1,07E-02	NS	NS	NS
**27**	72045*	72397*	352	No difference	4 bp	4 bp	LSD079	NS	NS	3,08E-02	NS	NS	NS

Recombination Sequence: RUSSIA /Saratov /2017

Major Parental Sequence(s): Vaccine = SIS-Lumpyvax (KX764643), Herbivac (KX764644), OBP (KX764645) vaccine and isolate Cro2016 (MG972412).

Major Parent = Parental strain contributing the larger fraction of sequence.

Minor Parental Sequence(s): Field = Kenya KSGP0240 (KX683219), Kenya NI-2490 (AF325528), RSA Warmbaths (AF409137), Greece Evros/GR15 (KY829023) and Serbia/Bukanovac/2016 (KY702007).

Minor Parent = Parental strain contributing the smaller fraction of sequence.

* = The actual breakpoint position is indeterminate (it was most likely obscured by a subsequent recombination event).

NS = No significant P-value was recorded for this recombination event using this method.

The majority of the predicted events lists the genome of LSDV RUSSIA/Saratov/2017 as the recombinant sequences being derived from the field viruses as the minor parent within the genetic backbone of the vaccine-derived viruses as the major parent. The percentage sequence identity shared between the complete genome of RUSSIA/Saratov/2017 was compared with SIS-Lumpyvax_vaccine (KX764643) virus representing the vaccine-related viruses in black and Bujanovac/Serbia/2016 (KY702007) representing the wild-type field isolates in grey using SimPlot (Fig 4). The graphical representation of the percentage similarities between these three virus genomes, support the RDP in that the RUSSIA/Saratov/2017 has the genetic backbone of a vaccine-like virus (100% similarity), with intermittent regions where it has a higher percentage sequence similarity to the wild-type field isolates than the vaccine-like viruses and that these regions correspond with the predicted RDP events (Fig 4). The first recombination event predicted the intragenic exchange of a region containing LSD144 and LSD145, which encode a Kelch-like and Ankyrin repeat protein respectively (Table 1 and Fig 4). This exchange of field virus sequence into the vaccine-related backbone was responsible for eight amino acid changes in LSD144, including the change from the frameshift truncated reading frames of LW144a and LW144b to the single ORF of LSD144 (Table 2). In addition, 26 amino acid exchanges in LSD145 were observed compared to the vaccine viruses, with the majority of these occurring in either one of the seven Ankyrin repeat domains or the C-terminal F-box. A second Kelch-like protein (LSD019) and a second Ankyrin repeat protein (LSD147) were involved in predicted recombination events (20 and 14) respectively, resulting in the exchange of three amino acids from field viruses into the vaccine backbone of LSD019, but no amino acid exchange occurred in LSD147 (Table 2 and Fig 4). Twelve proteins likely involved in RNA transcription, translation or modification have been predicted to be affected by recombination events (LSD084, LSD085, LSD086, LSD087, LSD088, LSD089, LSD049, LSD051, LSD055, LSD098, LSD071 and LSD110 in events 5, 7, 11, 13 and 16) (Table 1 and Fig 4). The most significant changes were observed in the two mutt motif proteins (LSD086 and LSD087 in recombination event 5), where the C-terminal of the proteins were changed to resemble the wild-type field viruses (Table 2). An additional five proteins (LSD135, LSD138; LSD008; LSD140 and LSD141) predicted to play a role in viral virulence and host range determination have been indicated to be involved in homologous recombination events (2, 3 and 12) (Table 1 and Fig 4). Both LSD135 and LSD138 had amino acid exchanges in their immunoglobulin domains reflecting the wild-type viruses, while no substitutions in the transmembrane segment of LSD138 were observed (Table 2). Three proteins involved in DNA replication (LSD133, LSD139 and LSD082 in events 4, 12 and 15, respectively), resulted in amino acid exchanges in the Uracil-DNA glycosylase domain of LSD082 and the protein kinase domain of LSD139 (Table 2). Twelve proteins involved in virus structure and assembly (LSD050, LSD053, LSD057; LSD060, LSD063, LSD102, LSD103, LSD104, LSD105, LSD141, LSD072 and LSD081 in events 7, 9, 10, 12, 13 and 15, respectively) were predicted to exchange DNA similar to the field viruses into the vaccine-related backbone (Table 1 and Fig 4).

**Table 2:** Proteins affected by the 27 recombination events. The predicted proteins are listed, as well as their putative functions. Predicted protein names are in bold if the complete ORF forms part of the recombination region. The number of amino acid differences between the predicted proteins of the minor and major parent are listed for the complete ORF. All the amino acid substitutions between the recombinant virus and the major parent groups are indicated along with their positions in the protein.

Recom- bination Event Number	Protein(s)	Amino acid differences (Minor and major parent)	Amino acid differences (Recombinant to major parent)
**1**	LSD144 (LW144a and LW144b) (Kelch-like protein); LSD145 (Ankyrin repeat protein)	LSD144: 36 aa; LSD145: 44 aa;	LSD144: 218 I>D = N; 234 S>N; 338; Translation identical to minor parent, thus 252–267 is FIYISDPNWYSKKYDI; 337^338 del>E; 375 Y>E; 385 D>S; 423 S>T; 456 E>A; 529 S>A; 548 terminate similar to major parent; LSD145: 8 I>V; 22 I>L; 121 S>G; 139 I>V; 144 V>I; 181 T>I; 189 F>L = L; 196 G>N; 202 S>R; 205 N>D; 210 T>S; 216 C>S; 241 K>Q; 261 M>V; 269 L>I; 273 N>S; 292 S>N; 309 S>P; 336 V>I; 340 I>V; 341 E>D; 342 H>N; 349 D>E; 353 Y>H;
**2**	LSD134 (LW134a and LW134b); LSD135 (putative IFN-alpha/beta binding protein); LSD136 (hypothetical protein); LSD137 (hypothetical protein); LSD138 (IG domain OX-2-like protein)	LSD134: 81 aa; LSD135: 7 aa; LSD136: 3 aa; LSD137: 4 aa; LSD138: 6 aa;	LSD134: 2 G>K; Translation similar to minor parent, thus 720–783 is QSLRRYSSSDYETIGENIYESIREPEYALLSKPRVLNPRSHIPLPSVPKDDIPFTQQKRKVID; 886 S>N; 1056 K>R = R; 1900 S>A; 2007^2008 del>N; 2018 N>del; LSD135: 11 L>S; 65 T>K; 129 A>T; 156 R>Q; 213 I>L; 249 S>P; 346 R>Y; LSD136: 32 V>I; 76 S>L; 80 N>D = D; 141 V>I; LSD137: 58 H>P; 106 V>I; 143 T>A; 212 S>A; LSD138: 14 G>S; 24 S>T; 43 S>N; 93 V>I; 100 Q>K;
**3**	LSD008 (Putative soluble interferon gamma receptor) LSD009 (Putative alpha amanitin-sensitive protein)	LSD008: 20 aa; LSD009: 13 aa;	LSD008: 16 S>Y; 32 N>K; 34 G>E; 36 I>T; 39 D>V; 40 N>S; 41 S>D; 45 K>R; LSD009: 129 R>K; 135 T>A; 142 T>A; 147 Y>H; 151 D>E; 166 H>R; 179 I>V; 185 I>M; 187 C>Y; 188 M>I; 200 K>T; 201 G>C;
**4**	LSD131 (Superoxide dismutase-like protein); **LSD132** (hypothetical protein); **LSD133** (DNA ligase-like protein); LSD134 (LW134a) (Similar to variola virus B22R)	LSD131: (53 aa truncation); **LSD132**: 5 aa; **LSD133**: 8 aa;	LSD131: C-terminal identical to minor parent, thus 84–161 is FNNERHIGDLGNIYSNKYGISYIYILDGKISLVGDYSIIGRSLVISEKNDDLGKGYNFKSFIDGNSGNGVAYGIIGIA; **LSD132**: 19 V>L; 63^64 del> T; 69 L>V; 98 T>N; 145 G>D; **LSD133**: 165 V>I; 275 S>N; 312 L>S; 446 N>K; 514 S>A;
**5**	LSD084 (Putative early transcription factor small subunit); **LSD085** (RNA polymerase subunit); **LSD086** (mutT motif protein); **LSD087** (mutT motif protein); **LSD088** (putative transcription termination factor); LSD089 (mRNA capping enzyme small subunit)	LSD084: 2 aa; **LSD085**: 1 aa; **LSD086**: 9 aa; **LSD087**: 2 aa and 53 aa C-terminal truncation; **LSD088**: 1 aa; LSD089: 2 aa;	LSD084: 353 L>G; 581 D>N; **LSD085**: 136 M>T; **LSD086**: 191 L>F; Translation similar to minor parent, thus 207–213 NTLVNSK; **LSD087**: 12 D>G; 46 V>I; C-terminal similar to minor parent, thus 199–253 SNKEIKSLVFFDSLYNGIEGDIIRFVLDISRLKCFGNKGYELYNKNTFKSLKSFF; **LSD088**: 24 V>I; LSD089: Identical to major parent;
**6**	LSD154 (putative ER-localized apoptosis regulator); **LSD155** (hypothetical protein); **LSD156** (hypothetical protein)	**LSD155**: No difference; **LSD156**: 2 aa	**LSD155**: 36 I>N = N; 43 G>V = V; 80 S>T = T; 81 R>S = S; **LSD156**: 92 F>Y = Y; 136 F>I = I; 144 I>M;
**7**	LSD049 (putative RNA helicase); **LSD050** (putative metalloprotease); **LSD051** (putative transcriptional elongation factor); **LSD052** (hypothetical protein); **LSD053** (putative glutaredoxin); **LSD054** (hypothetical protein); **LSD055** (Similar to G5.5R, RPO7); **LSD056** (hypothetical protein); LSD057 (putative virion core protein)	LSD049: 5 aa; **LSD050**: 1 aa; **LSD051**: No differences; **LSD052**: 1 aa; **LSD053**: No differences; **LSD054**: 3 aa; **LSD055**: No differences; **LSD056**: 1 aa; LSD057: 2 aa	LSD049: 145 A>T; 623 K>R; 673 V>I; **LSD050**: Identical to major parent; **LSD051**: Identical to both parents; **LSD052**: 33 K>N; **LSD053**: Identical to both parents; **LSD054**: 54 S>G; 184 M>I; **LSD055**: Identical to both parents; **LSD056**: 171 K>R = R; 174 N>D; LSD057: 257 V>A; 372 V>I;
**8**	LSD035 (LW036) (hypothetical protein); **LSD037** (hypothetical protein); **LSD038** (hypothetical protein); LSD039 (DNA polymerase)	**LSD037**: 4 aa; **LSD038**: No differences; LSD039: 8 aa	**LSD037**: 251 V>F; 300 V>I; 437 I>V; 564 G>V; **LSD038**: Identical to both parents; LSD039: Identical to major parent;
**9**	LSD059 (putative myrostylated protein); **LSD060** (putative myristylated IMV envelope protein); **LSD061** (hypothetical protein); **LSD062** (hypothetical protein); LSD063 (putative DNA-binding virion core protein)	LSD059: 1 aa; **LSD060**: No differences; **LSD061**: 3 aa; **LSD062**: 2 aa; LSD063: No differences	LSD059: Identical to major parent; **LSD060**: Identical to both parents; **LSD061**: 13 D>E; 82 L >S = A; **LSD062**: 185 R>K; 273 I>V; LSD063: Identical to both parents;
**10**	LSD102 (hypothetical protein); **LSD103** (putative virion core protein); **LSD104** (putative IMV membrane protein); **LSD105** (putative IMV membrane protein); LSD106 (putative virulence factor);	LSD102: 3 aa; **LSD103**: 2 aa; **LSD104**: No differences; **LSD105**: No differences; LSD106: No differences	LSD102: Identical to major parent; **LSD103**: 50 T>N; 72 P>T; 89 S>G = G; **LSD104**: Identical to both parents; **LSD105**: Identical to both parents; LSD106: Identical to both parents;
**11**	LSD097 (hypothetical protein); LSD098 (putative early transcription factor large subunit);	LSD097: 2 aa; LSD098: 6 aa;	LSD097: 25 V>A; 65 L>V; LSD098: 652 T>I;
**12**	LSD139 (putative Ser/Thr protein kinase); **LSD140** (putative RING finger host range protein); LSD141 (putative EEV host range protein);	LSD139: 5 aa; **LSD140**: 10 aa; LSD141: 4 aa;	LSD139: 263 G>V; 272 S>P; **LSD140**: 5 S>I; 132 T>M>K; 187 K>N; 228 N>D; LSD141: 38 E>K
**13**	LSD071 (RNA polymerase subunit); **LSD072** (putative protein-tyrosine phosphatase); LSD073 (hypothetical protein)	LSD071: 4 aa; **LSD072**: No differences; LSD073: 2 aa;	LSD071: 879 N>S; **LSD072**: Identical to both parents; LSD073: 26 T>S
**14**	LSD147 (ankyrin repeat protein)	LSD147: 1 aa;	LSD147: Identical to major parent;
**15**	LSD081 (putative virion protein); LSD082 (uracil DNA glycosylase);	LSD081: 2 aa; LSD082: 1 aa;	LSD081: 227 S>N; LSD082: 54 R>Q
**16**	LSD110 (putative DNA helicase transcriptional elongation factor); LSD111 (hypothetical protein)	LSD110: 7 aa; LSD111: No differences;	LSD010: Identical to major parent; LSD111: Identical to both parents;
**17**	LSD108 (putative myristylated membrane protein)	LSD108: 3 aa;	LSD108: 171 S>A;
**18**	LSD023 (hypothetical protein)	LSD023: No differences;	LSD023: Identical to both parents;
**19**	LSD034 (putative PKR inhibitor); LSD036 (LW035a) (RNA polymerase subunit);	LSD034: 1 aa; LSD036 (LW035a): 1 aa;	LSD034: Identical to major parent; LSD035: P>S = S; LSD036: Identical to major parent;
**20**	LSD019 (kelch-like protein)	LSD019: 4 aa and a 150 aa N-terminal truncation	Identical frameshift to LW019a and LW019b. LSD019: 84 A>V; 89 K>R; 104 V>A;
**21**	LSD075 (RNA polymerase-associated protein)	LSD075: 5 aa;	LSD075: 692 S>N; 739 F>L;
**22**	LSD091 (putative late transcription factor); LSD092 (putative late transcription factor);	**LSD091**: No differences; LSD092: No differences;	**LSD091**: Identical to both parents; LSD092: Identical to both parents;
**23**	LSD027 (putative EEV maturation protein);	LSD027: 6 aa	LSD027: 327 D>N;
**24**	LSD042 (hypothetical protein)	LSD042: 4 aa;	LSD042: 190 A>T; 230 P>S;
**25**	LSD002 (hypothetical protein); LSD003 (putative ER-localized apoptosis regulator;	LSD002: No differences; LSD003: 2 aa;	LSD002: Identical to both parents; LSD003: Identical to major parent;
**26**	LSD020 (ribonuclease reductase small subunit); LSD021 (hypothetical protein)	LSD020: 3 aa; LSD021: 3 aa;	LSD020: Identical to major parent; LSD21: 78 T>K;
**27**	LSD079 (mRNA capping enzyme large subunit)	LSD079: 4 aa;	LSD079: Identical to major parent;

Approximately 156 to 159 ORFs are predicted for LSDV, accounting for 48,000 amino acids. There are 1,436 amino acid differences, including 889 gaps, when comparing LSDV from the wild-type field isolate group to the live attenuated vaccine or vaccine-derived group (Fig 3). This implies that the majority of the over 2000 nucleotide differences between these groups are nonsynonymous, yet the amino acid exchanges in Table 2 indicated that these are primarily conserved (like–for-like). A significant impact on the resulting predicted proteins is evident when the surmised recombination events were responsible for altering the translation of the ORFs. These changes were observed in ORFs LSD086, LSD087, LSD134 and LSD144, where the ORFs in the vaccine or vaccine-derived viruses had premature translation termination codons in comparison to the wild-type field viruses, while these ORFs of virus RUSSIA/Saratov/2017 abolished the termination codons resulting in a full-length proteins being generated. Additionally, a shift in the reading frame is observed in LSD131 of virus RUSSIA/Saratov/2017, which also abolished the stop codon to produce a new C-terminal. Each of these recombination events of virus RUSSIA/Saratov/2017 resulted in a complete protein, similar to the field viruses, rather than the truncations observed in the vaccine viruses (Table 2).

## Discussion

Lumpy skin disease virus was originally described and isolated in southern Africa as the causative agent for a synonymous disease, From which the disease spread rapidly northwards throughout Africa and into the Middle East, where from 2014–2017 it was first reported in Turkey, eastern Europe and Russia [14, 30].

A live-attenuated vaccine was developed in the 1960s in South Africa from a Neethling-type field strain, first by multiple passage in lamb kidney cells, then on the chorioallantoic membranes of embryonated hen’s eggs and finally, again in cell culture [31]. Importantly, this method of attenuation, used in developing this vaccine and other LSD vaccines in use today, generates relatively random mutations throughout the viral genome due to the altered selection pressures [32], without characterisation of these mutations until recently [33,34]. Achieving the perfect balance between attenuation (no severe post-vaccinal reactions) and immunogenicity using this method is challenging, with only mild post-vaccinal reactions and good levels of immunity experienced in general in cattle in LSD endemic regions when vaccinated with these vaccines, but with more severe reactions being experienced in naïve cattle populations [35, 36]. This observation suggests that a number of the genetic differences identified in the recently sequenced LSDV vaccine strains may still be insufficient in reducing the overall virulence of the virus. This was highly evident in the use of one or more of these vaccines recently in Eastern Europe whereby adverse clinical signs were induced, including skin lesions, viral shedding in nasal discharges and viremia [36]. More recently, two new vaccines were developed and commercialized in South Africa, the sequences of which have a high percentage of sequence similarity to the original South African LSD vaccine [33]. Such live vaccines were actively administered during the 2015–2017 outbreaks in south-eastern Europe and Kazakhstan [5]. Viraemic animals with live virus shed in skin scabs and nasal discharges were never considered as a threat to unvaccinated animals for several reasons. Firstly, no conclusive evidence has yet been obtained pertaining to the mode (or modes) of transmission of LSDV, negating any risks of vaccine escape. Secondly, predicting the potential for vaccine genetic alterations leading to reversion to virulence due to recombination with a virulent field strain was impossible due to the lack of sequencing data and molecular tools to differentiate between the two, which only became available relatively recently [15,16]. In general, recombination drives genetic diversity and viral evolution. Up to now, evidence of recombination events have been described for various DNA and RNA viruses, but with different frequencies and efficiencies [37–39]. The rates of recombination are influenced by the selection pressures exerted on the virus population and contribute to virus evolution through genetic shift. In order for homologous intragenic recombination to occur, it is essential that a single cell, either in cell culture, a host or vector, is co-infected by two or more viruses with homologous genomes. This could occur when live attenuated vaccine viruses are administered during an outbreak in which field strains are circulating [13].

Live attenuated vaccines against LSD are not permitted for use within the territory of the Russian Federation, but are authorized for use in the north of Kazakhstan, bordering with Russia in the south. Since the start of the vaccination campaign, there were concerns that a live vaccine virus would escape and spill into cattle of a neighboring country. The first case of vaccine-like LSDV strain detection was reported in 2017 in a Russian region sharing its border with regions of Kazakhstan in which vaccination of cattle occurred [6].

In this paper, we report on follow-up studies on our findings obtained from 2017 in Russia [6] and attempt to gain better insight into the events leading to generation of a recombinant vaccine-like LSDV strain recovered from an affected backyard cow exhibiting clinical signs of LSD using full genome sequencing to characterise the virus.

The LSDV RUSSIA/Saratov/2017 strain was isolated around 60 km from the Kazakhstan border, successfully cultured and sequenced using an Illumina platform (USA). It was identified as having vaccine components using the GPCR PCR [18] and field components using the RPO30 PCR [17] (Figs 1 and 2). Genomic analysis showed that it compares well with both field and vaccine virus strain sequences deposited in GenBank in terms of length (nt) and number of ORFs (a total of 158 ORFs). Herein, we identified 27 possible homologous intragenic recombination events within its genome. These 27 events were supported by significant statistical analyses determined using either GENECONV, Bootscan or RDP algorithms [28]. Although an additional nine events were predicted, they were discarded due to low statistical support using the aforementioned algorithms in RDP. The RDP-assisted analysis showed that the recombinant LSDV RUSSIA/Saratov/2017 strain has a genomic backbone similar to the three live attenuated virus strains commercially available as LSD vaccines included in this study. Since the genomes of all three strains share 99.9% homology with each other, we were unable to determine the identity of the LSD vaccine from which the recombinant Russian strain’s backbone originated. The minor parents or donors of the 27 predicted recombination events were attributable to any one of the five sequenced field LSDV strains, the full genome sequences of which are publically available in GenBank.

Phylogenetic analysis of the full genome sequences placed the strain between the field and vaccine groups (Fig 4) with significant bootstrap support. Of note, comparative analysis of whole-genome sequences of field and vaccine LSDV strains clearly demonstrate that the genome of LSDV is highly conserved. The results of a number of studies investigating recombination events within the family *Poxviridae* have been published to date. Lin and Evans (2010) discovered that upon coinfection vaccinia viruses experience intracellular constraints to recombination, although the process is possible [40]. Li Qin et al (2011) analysed vaccinia virus strain variants in a vaccine preparation and discovered a complex mixture of hybrid viruses displaying a random pattern of recombination events across the genomes [41]. Unfortunately, evidence for these occurrences is scarce and mainly limited to orthopoxviruses. Attempts were made in the past to speculate about evolution of capripoxviruses, however, to date no intra-strain live recombinant viruses within the genus have been obtained naturally nor been generated *in vitro* [42].

Herein, we report the identification of a likely vaccine escape recombinant strain of LSDV predicted to have been derived from a Neethling-type vaccine strain and a field strain. Since this virus was recovered from a host (cow) displaying severe clinical signs, either the vaccine escape recombinant demonstrates an evolutionary reversal of attenuation or a field strain was also present which induced the clinical signs and which escaped detection and gave rise to such a novel strain [37].

Importantly, the LSDV RUSSIA/Saratov/2017 strain is more closely genetically related to the vaccine strains compared in this study (>99.3%), than the field isolates (>98.64%). A detailed analysis of its genome indicates the presence of a vaccine virus-derived backbone with field virus-derived sequences interspersed throughout the genome. The average distance between the recombination sites is around 3400 bps. It is evident that the recombination events predicted in this study were not random and appear to have been under the influence of selection pressure. Nine frameshifts have been described between the LSDV Warmbaths and OBP vaccine strain, resulting in truncations of the expressed proteins [42]. Through the process of recombination, five of these ORFs (LSD086, LSD087, LSD131, LSD143 and LSD144) were restored to full-length ORFs in virus strain LSDV RUSSIA/Saratov/2017 to resemble the wild-type virulent field viruses. The emergent virus sheds light on our understanding of capripoxvirus genetics. The recombination that took place in a host co-infected with a vaccine and field strain had possibly been precipitated by a massive spread of one ore more vaccine strains of LSDV in at least Saratovskaya oblast [43]. Moreover, not only in Saratovskaya oblast, but also in other regions of Russia bordering on Kazakhstan where “vaccine” strains of LSDV were documented in 2017 and 2018 (unpublished data). The occurrence of vaccine LSDV strains in Russian cattle can only be explained by virus escape (or, highly risky and illegal use of LSD vaccine). However, since the genetic background critical for the attenuation of LSDV is still poorly understood, the difference in transmission efficiency between field and vaccine strains of LSDV needs to be assessed under control conditions.

The current findings raise crucial issues concerning the vaccinology concept of DIVA (differentiating infected from vaccinated animals) and the accuracy of phylogenetic analysis. First, the novel virus characterized in this study can no longer be considered vaccine as it greatly differs from the one administered to animals in terms of its genetic backbone and phenotype. There are a number of real-time PCR assays published that could be utilized to identify the strain genotype. The Agianniotaki assay [16] employs the GPCR target to distinguish between the two genotypes. According to their assay, the LSDV RUSSIA/Saratov/2017 strain tests as vaccine because it carries a vaccine locus of GPCR, which is not correct. The assay by Menasherow et al [44] differentiates LSDVs based on the 27 bp insertion/deletion in the EEV gene (ORF 126) and thus the recovered strain in this study would come up as vaccine. The combination of the vaccine assay, based on LSDV 005 [6], and the field starin assays by Pestova et al [19] and Vidanovic et al [45], targeting the 27 bp insertion, would also produce negative results since the targeted loci are reversed. The hazard of spreading of such a “vaccine” strain and causing severe clinical signs, and not being reported to OIE (vaccine strains which are detected are exempt from notification following vaccination) is multiplied in this scenario.

As for phylogeny, single-locus typing has always been the most convenient method for tree inference, however, the emergence of recombinant viruses worldwide necessitates the use of more than one locus for investigating the potential for recombination.

The results of this study are important in that they provide clear evidence for natural recombination occurring between field and vaccine strains of capripoxvirus. The true significance of the results are yet to be realized, pertaining to present and future control of LSD and use of live attenuated vaccines during active outbreaks. Reports of “vaccine failure” [33] and “vaccine disease” need to be investigated in the context of the genetic evidence presented herein to ascertain whether such events are more common than previously thought, possibly leading to new virus strains with altered virulence properties (compare with studies on myxoma virus within Europe and Australia [46]). What is almost certain is that LSD will continue to spread and vaccination has been shown to be the only effective method of prevention and control of large-scale outbreaks—the challenge will be to ensure that effective coverage is maintained across man-imposed boundaries, using vaccines which are safe and efficacious.
